# Influence of noise-binding energy interplay on the second and third-order nonlinear optical properties of impurity doped quantum dots

**DOI:** 10.1016/j.heliyon.2019.e01785

**Published:** 2019-06-01

**Authors:** Anuja Ghosh, Sk. Md. Arif, Manas Ghosh

**Affiliations:** Department of Chemistry, Physical Chemistry Section, Visva-Bharati University, Santiniketan, Birbhum 731235, West Bengal, India

**Keywords:** Materials science, Quantum dot, Impurity, Binding energy, Second and third-order nonlinear optical properties, Gaussian white noise

## Abstract

We inspect the role of binding energy (BE) on second-order and third-order nonlinear optical (NLO) properties of doped GaAs quantum dot (QD). In the study ample stress is given on understanding the role of noise on the manifestations of these NLO properties. The profiles of these NLO properties are analyzed mainly on the basis of variation of two important criteria viz. peak-shift and peak-height as a function of BE. Both these features depend on the presence of noise, its pathway (mode) of introduction and sometimes on the identity of the NLO properties. The findings of the study deem significance in realizing the binding energy-dependence of the said NLO properties of low-dimensional semiconductor materials when noise contribution becomes noticeable.

## 1 Introduction

Tremendous enhancement in the research on low-dimensional semiconductor systems (LDSS) e.g. quantum wells (QWLs), quantum wires (QWRs) and quantum dots (QDs) has been envisaged over the last couple of decades. The said enhancement can be justified from two different but highly related angles; technological and pedagogical. The technological angle deals with the widespread application of LDSS in the manufacture of high-output microelectronic and optoelectronic devices such as QD lasers, solar cells, single electron transistors and quantum computers. Simultaneously, the pedagogical angle involves rejuvenation of many fundamental concepts of quantum mechanics. Sudden rise in the level of delicacy of LDSS physics occurs with the introduction of impurity (dopant). The increased delicacy has its roots in the newly formed interaction between the original LDSS confinement potential and the dopant potential. The resultant effective confinement potential discernibly modifies the nonlinear optical (NLO) properties of LDSS from that of a dopant-free condition. LDSS possess special status for displaying enhanced NLO properties. Thus, the modulation of NLO properties of LDSS by impurity is quite interesting and accentuates rigorous research works [1], [2], [3], [4], [5], [6], [7], [8], [9], [10], [11], [12], [13], [14], [15], [16], [17], [18], [19], [20], [21], [22], [23], [24], [25], [26], [27], [28], [29], [30], [31], [32], [33], [34], [35], [36].

Presence of *noise* markedly affects the efficacy of LDSS-based devices. Noise can make its entry to the system by using some external ‘modes’ or ‘pathways’, among which *additive* and *multiplicative* are the two most common ones. These two pathways can be distinguished depending on how noise gets engaged with the system coordinates. Noise, thus introduced, conspicuously changes the physical properties of the system through the alteration of the effective confinement potential. Importantly, such change in the physical properties of LDSS has been found to be dependent on the mode of introduction of noise mentioned before. Therefore, exploration of noise-induced modulations of the physical properties of LDSS draws sincere attention.

Recently we have studied the influence of noise-binding energy interplay on a magnetic property viz. diamagnetic susceptibility (DMS) of doped QDs [37]. Current study endeavors to carry out an in-depth scrutiny of how the *interplay between noise and binding energy (BE)* can fine-tune three important NLO properties of 2-d GaAs QD viz. *electro-optical effect (EOE)* [38], [39], [40], *third-order nonlinear optical susceptibilities (TONOS)* [41], [42], [43], [44], [45], [46], [47], [48], [49], [50], [51] and *total optical dielectric function (TODF)* [52]. Thus, the present study shows some new innovation with respect to our previous study [37]. Among the mentioned NLO properties EOE and TONOS stand for the second-order and third-order nonlinear phenomena, respectively. TODF deserves importance since an extended inspection which commences from it culminates into understanding the effective optical properties of the dot-matrix composite systems because of dielectric mismatch. In this context BE of LDSS deems importance as any alteration in BE noticeably affects the physical properties of LDSS, including the NLO properties [5], [13], [19]. Thus, such study appears significant even from a technological viewpoint. In the present work the x−y confinement is described by harmonic oscillator potential and the *z*-confinement is offered by a vertical magnetic field. In addition, an electric field of strength *F* is applied to the system along *x* and *y*-directions. The QD is doped with *Gaussian impurity* and at the same time is subjected to *Gaussian white noise* applied via *additive* and *multiplicative* pathways (modes). The study makes a close scrutiny of how the interplay between BE and noise engineers the above NLO properties with substantial thrust on the influence of the noise mode.

## 2 Methods

The system delineated above may be expressed by the Hamiltonian ($H_{0}$):

(1) $$H_{0}=H_{0}^{\prime }+V_{imp}+|e|F(x+y)+V_{noise}.$$

$H_{0}^{\prime }$ is the impurity-free Hamiltonian and *e* is the electronic charge. Use of effective mass approximation leads to

(2) $$H_{0}^{\prime }=\frac{1}{2m^{⁎}}{\left[-i\hbar \nabla +\frac{e}{c}\text{A}\right]}^{2}+\frac{1}{2}m^{⁎}\omega_{0}^{2}(x^{2}+y^{2}).$$

$m^{⁎}$ and $\omega_{0}$ represent the effective mass of the electron and the harmonic confinement frequency, respectively. The vector potential **A** is given by A=(By,0,0), where *B* is the strength of the magnetic field. $H_{0}^{\prime }$ can be alternatively written as

(3) $$H_{0}^{\prime }=-\frac{\hbar^{2}}{2m^{⁎}}\left(\frac{\partial^{2}}{\partial x^{2}}+\frac{\partial^{2}}{\partial y^{2}}\right)+\frac{1}{2}m^{⁎}\omega_{0}^{2}x^{2}+\frac{1}{2}m^{⁎}\Omega^{2}y^{2}-i\hbar \omega_{c}y\frac{\partial }{\partial x}.$$

$\Omega (=\sqrt{\omega_{0}^{2}+\omega_{c}^{2}})$ and $\omega_{c}(=\frac{eB}{m^{⁎}c})$ are the effective confinement frequency in the *y*-direction and the cyclotron frequency, respectively.

$V_{imp}$ stands for the impurity (dopant) potential and has the form $V_{imp}=V_{0}\;e^{-\gamma \left[{\left(x-x_{0}\right)}^{2}+{\left(y-y_{0}\right)}^{2}\right]}$. Here $\left(x_{0},y_{0}\right)$, $V_{0}$ and $\gamma^{-1/2}$ refer to the dopant location, impurity strength and the available space under the influence of impurity potential, respectively. The use of such Gaussian impurity potential as a modification of the confinement potential was first introduced by Szafran et al. in the context of exciton spectrum of a quantum ring [53]. Such Gaussian impurity has also drawn the attention of several other researchers [54], [55], [56], [57], [58]. Adamowski studied the screening effect of the *LO* phonons of the $D^{-}$ center confined by a Gaussian potential QD, $V(r)=V_{0}\;exp[-{\left(\frac{r}{R}\right)}^{2}]$ [56]. Gaussian potential stands for the finite depth and range of QD confinement potential. Gaussian potential is a smooth potential and therefore is a good approximation to the impurity potential in electrostatic quantum dots [57], in which the spatial restriction originates from an inhomogeneous electric field. In self-assembled quantum dots with a composition modulation [59], the impurity potential can also be represented by the Gaussian potential [58]. Such Gaussian potentials can be assumed to simulate nanocrystals fabricated by means of colloidal chemical synthesis [60], [61], [62].

The quantity $V_{noise}$ of eqn. (1) takes care of the externally introduced Gaussian white noise characterized by *zero average* and *spatial δ-correlation*. Such application of noise to the system can be done in two separate modes (known as additive and multiplicative) which indeed govern the system-noise interaction. Mathematically speaking, noise involves a spatially *δ*-correlated function [f(x,y)] which assumes a Gaussian distribution (produced by Box-Muller algorithm) having strength *ζ* and is described by the set of conditions

(4) 〈f(x,y)〉=0,

the zero average condition, and

(5) $$\langle f(x,y)f(x^{\prime },y^{\prime })\rangle =2\zeta \delta \left[(x,y)-(x^{\prime },y^{\prime })\right],$$

the spatial *δ*-correlation condition. The additive and multiplicative pathways of introduction of noise can be written as

(6) $$V_{noise}=\lambda_{1}f(x,y),$$

for additive pathway and

(7) $$V_{noise}=\lambda_{2}f(x,y)(x+y),$$

for the multiplicative pathway. $\lambda_{1}$ and $\lambda_{2}$ are two arbitrary parameters in case of additive and multiplicative noise, respectively. In reality, there exist a variety of physical situations in which external noise can be realized and bears interest. In these situations one deals with system which experiences fluctuations which are not *self-originating*. These fluctuations can be due to a fluctuating environment or can be consequence of an externally applied random force. Whereas additive noise does not interfere with the system coordinate the multiplicative analogue depends on the instantaneous value of the variables of the system. It does not scale with system size and is not necessarily small [63], [64]. We can regard the external noise as an external field which drives the system [64]. Experimentally, external noise can be generated by using a function generator (Hewlett-Packard 33120A) and its characteristics, viz. Gaussian distribution and zero mean can be maintained [65]. The external noise could be introduced multiplicatively using a circuit that enables to drive the nonlinear element by using the voltage from an external source [66].

Now, the construction of Hamiltonian matrix ($H_{0}$) [cf. eqn. (1)] has been carried out using the direct product basis of the harmonic oscillator eigenstates. The matrix elements corresponding to all the four components of eqn. (1) have been derived using the basis function mentioned above. It is followed by diagonalization of $H_{0}$ to compute the energy levels and the eigenstates of the system. The routine convergence test has been done during diagonalization.

For the computation of different NLO properties it is required to consider an ensemble of QDs and how it interacts with a polarized monochromatic electromagnetic field of angular frequency *ω*. Such an analysis implicitly assumes that the wavelength of propagating electromagnetic wave is higher than the QD size. Driven by this assumption we can envisage the wave with nearly unaltered amplitude along QD and the electric dipole approximation gets justified. Now, exploiting customary density matrix approach and iterative procedure the expressions of EOE [39], TONOS [43], [44], [45] and TODF [52] can be obtained under appropriate conditions [67].

Thus, the expression of EOE coefficient is given by [39]

(8) $$\chi_{EOE}=\frac{8e^{3}\sigma_{s}}{n_{r}^{4}\varepsilon_{0}^{3}\hbar^{2}}M_{ij}^{2}\delta_{ij}.\frac{\nu^{2}\Gamma^{4}}{\left[{\left(\omega_{ij}-\nu \right)}^{2}+\Gamma^{2}\right].\left[{\left(\omega_{ij}+\nu \right)}^{2}+\Gamma^{2}\right]}.$$

In the above expression $\varepsilon_{0}$ is the vacuum permittivity, *e* is the absolute value of electron charge, $\sigma_{s}$ is the carrier density, $M_{ij}=e\langle \psi_{i}|\overset{ˆ}{x}+\overset{ˆ}{y}|\psi_{j}\rangle ,(i,j=1,2)$ is the matrix elements of the dipole moment, $\delta_{ij}=|M_{ii}-M_{jj}|$, $\psi_{i}(\psi_{j})$ are the eigenstates, $n_{r}$ is the static component of refractive index and $\omega_{ij}=(E_{i}-E_{j})/\hbar$ is the transition frequency, Γ=1/τ is the relaxation rate with *τ* as the relaxation time.

Using similar approach as stated above, within second-order perturbation theory, TONOS corresponding to optical mixing between two incident light beams with frequencies $\nu_{1}$ and $\nu_{2}$ is given by [43], [44], [45]

(9) $$\chi^{\left(3\right)}(-2\nu_{1}+\nu_{2};\nu_{1},\nu_{1},-\nu_{2})=\frac{-2ie^{4}\sigma_{s}M_{ij}^{4}}{\varepsilon_{0}\hbar^{3}\left[i(\omega_{ij}-2\nu_{1}+\nu_{2})+\Gamma \right].\left[i(\nu_{2}-\nu_{1})+\Gamma \right]}\times \left[\frac{1}{i(\omega_{ij}-\nu_{1})+\Gamma }+\frac{1}{i(\nu_{2}-\omega_{ij})+\Gamma }\right],$$

Pursuing Xie [43], [44], [45], in the present study we consider $\nu_{1}=-\nu_{2}=\nu$ for simplicity.

Following Vahdani, considering optical transition between two states $|\psi_{0}\rangle$ and $|\psi_{1}\rangle$, the linear [$\chi^{\left(1\right)}(\nu )$] and the third-order nonlinear [$\chi^{\left(3\right)}(\nu )$] electric susceptibilities can be written as [52]

(10) $$\chi^{\left(1\right)}(\nu )=\frac{\sigma_{s}|M_{01}{|}^{2}}{E_{01}-\hbar \nu -i\hbar \Gamma },$$

and

(11) $$\chi^{\left(3\right)}(\nu )=-\frac{\sigma_{s}|M_{01}{|}^{2}|\overset{˜}{E}{|}^{2}}{E_{01}-\hbar \nu -i\hbar \Gamma }.[\frac{4|M_{01}{|}^{2}}{{\left(E_{01}-\hbar \nu \right)}^{2}+{\left(\hbar \Gamma \right)}^{2}}-\frac{{\left(M_{11}-M_{00}\right)}^{2}}{\left(E_{01}-i\hbar \Gamma \right)\left(E_{01}-\hbar \nu -i\hbar \Gamma \right)}].$$

As stated by Vahdani, the linear and third-order nonlinear ODFs are related to $\chi^{\left(1\right)}(\nu )$ and $\chi^{\left(3\right)}(\nu )$ as follows [52]:

(12) $$\epsilon^{\left(1\right)}(\nu )=1+4\pi \chi^{\left(1\right)}(\nu ),$$

and

(13) $$\epsilon^{\left(3\right)}(\nu )=4\pi \chi^{\left(3\right)}(\nu ).$$

The TODF is given by

(14) $$\epsilon (\nu )=\epsilon^{\left(1\right)}(\nu )+\epsilon^{\left(3\right)}(\nu ).$$

The ground state binding energy $E_{B}$ can be written as

(15) $$E_{B}=E_{0}-E,$$

where *E* and $E_{0}$ are the energies of the ground state with and without impurity, respectively.

## 3 Results & discussion

We have used ε=12.4 and $m^{⁎}=0.067m_{0}$ ($m_{0}$ is the mass of free electron). The values of a few important quantities are kept fixed at: $\hbar \omega_{0}=250.0\text{ meV}$, $V_{0}=280.0\text{ meV}$, B=20.0 T, F=100 kV/cm, $r_{0}=0.0$ nm and $\zeta =1.0\times 10^{-4}$, where *ζ* is the noise strength. The BE value has been considered up to 200 meV [68], [69]. We also use the abbreviations ADN and MLN to represent additive noise and multiplicative noise, respectively.

**Figure 1:**
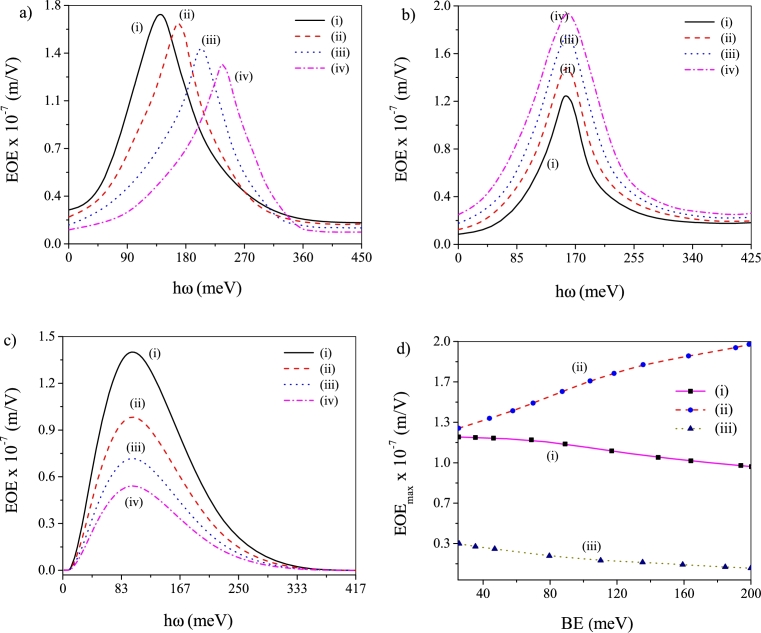
Plots of EOE against *hω* for four different values of BE: (a) noise-free state, (b) ADN operates and (c) MLN operates. In these plots (i) *BE* = 25 meV, (ii) *BE* = 75 meV, (iii) *BE* = 125 meV and (iv) *BE* = 200 meV. (d) Plot of EOE peak values vs BE: (i) noise-free state, (ii) ADN operates and (iii) MLN operates.

Fig. 1 depicts the change of EOE with energy of incoming photon (*hω*) for four different values of BE (25 meV, 75 meV, 125 meV and 200 meV) without noise [Fig. 1a] and when ADN [Fig. 1b] and MLN [Fig. 1c] are applied. Under a noise-free ambience a regularly increasing BE depletes the EOE peak height and causes *blue-shift* of EOE peak [39], [40]. Above observation indicates that an enhancement in BE augments the system confinement and quenches the spatial spread of the eigenstates. The amplified confinement eventually amplifies the energy intervals among the eigenstates and causes the *blue-shift* of EOE peaks. And the reduced spatial stretch of the eigenstates decreases the mutual overlap between them leading to drop in the EOE peak height. Thus, in absence of noise, low BE of the system is preferred for production of large EOE. Presence of noise (both ADN and MLN) results into *unshifted* EOE peak as BE increases. However, the peak height enhances (decreases) with BE when ADN (MLN) is introduced. It, therefore, becomes evident that high (low) BE of the system is appropriate for emergence of large EOE under applied ADN (MLN). The plot of peak values of EOE as a function of BE under different conditions [Fig. 1d] runs in conformity with above findings as it depicts a steady drop in the peak value with BE under noise-free state and when MLN is present. The same plot also evinces growth in the peak value with BE when ADN is present.

**Figure 2:**
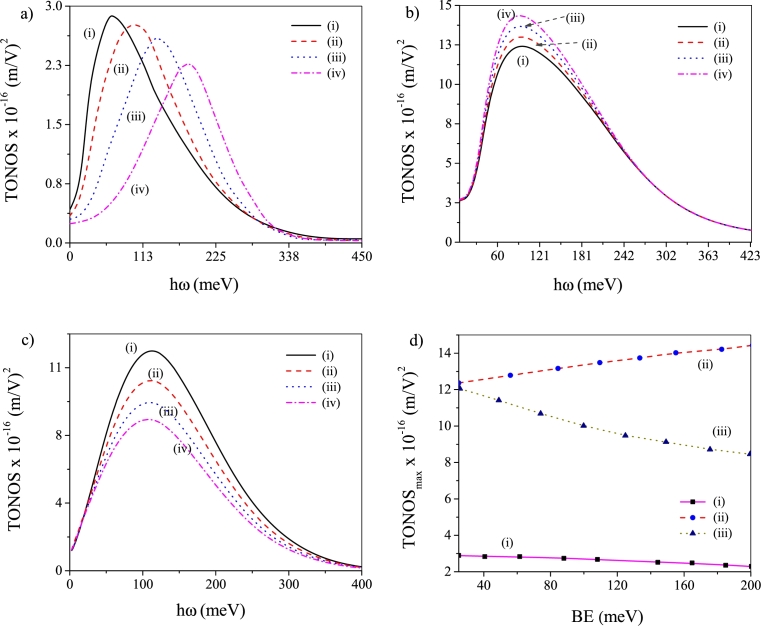
Plots of TONOS against *hω* for four different values of BE: (a) noise-free state, (b) ADN operates and (c) MLN operates. In these plots (i) *BE* = 25 meV, (ii) *BE* = 75 meV, (iii) *BE* = 125 meV and (iv) *BE* = 200 meV. (d) Plot of TONOS peak values vs BE: (i) noise-free state, (ii) ADN operates and (iii) MLN operates.

Figs. 2(a-d) depict the TONOS profiles relevant to BE change under different conditions. Under noise-free condition, the TONOS peaks display *blue-shift* and fall of peak height with gradual increase of BE of the system [Fig. 2a] [70]. It thus becomes evident that there occur amplification in the energy separation and reduction in the extent of mixing between the eigenfunctions as BE steadily increases. The said amplification originates from the increased quantum confinement effect that follows an increase in BE. The increased confinement also diminishes the spatial stretch of wave functions thereby decreasing the dipole transition matrix elements. The fall in the TONOS peak height with BE thus becomes obvious. Hence, without noise, a low BE appears to be conducive for emergence of large TONOS. Introduction of noise leads to diverse behavior in the variation of TONOS peak height with gradual enhancement of BE. The said diversity is linked with the mode of introduction of noise [Figs. 2(b-c)]. Introduction of ADN (MLN) leads to rise (fall) in the TONOS peak height with increase in BE. However, both under applied ADN and MLN the TONOS peaks remain nearly *unshifted* as BE increases. Thus, generation of large TONOS needs high (low) BE of the system when ADN (MLN) is applied. Above findings are corroborated by TONOS peak value vs BE [Fig. 2d] plot under various conditions. The plot reveals fall in the TONOS peak value with increase in BE without noise and when MLN is present. On contrary, TONOS peaks depict steady rise with increase in BE when ADN operates.

**Figure 3:**
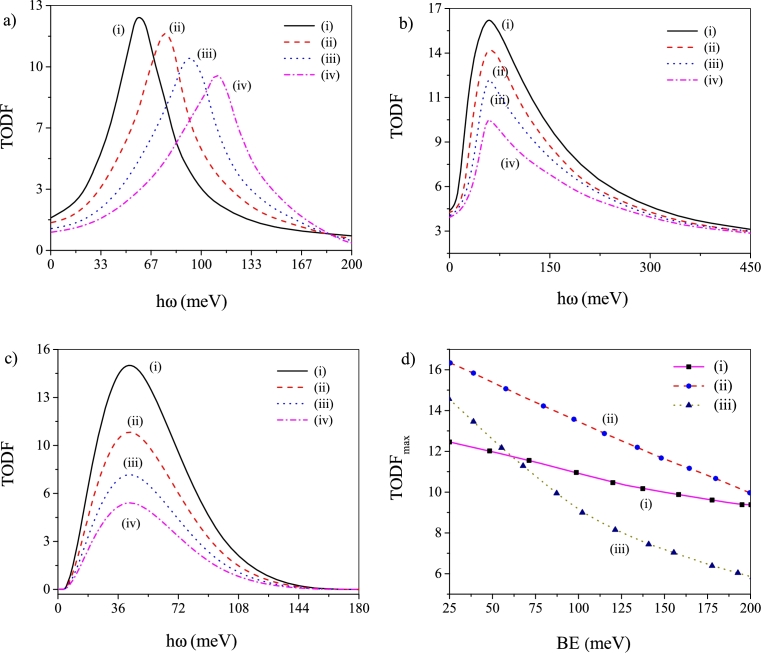
Plots of TODF against *hω* for four different values of BE: (a) noise-free state, (b) ADN operates and (c) MLN operates. In these plots (i) *BE* = 25 meV, (ii) *BE* = 75 meV, (iii) *BE* = 125 meV and (iv) *BE* = 200 meV. (d) Plot of TODF peak values vs BE: (i) noise-free state, (ii) ADN operates and (iii) MLN operates.

Figs. 3(a-d) exhibit the TODF profiles related to BE without noise effect [Fig. 3a] and when ADN [Fig. 3b] and MLN [Fig. 3c] are present. Under all circumstances the TODF peaks display prominent drop in the peak height as BE increases. The observation reflects enhanced confinement accompanying the increase in BE under different situations. The enhanced confinement forces the wave function to localize and depletes its overlapping ability manifested through drop in the TODF peak height. Thus, a small value of BE would be essential to generate large TODF under all conditions. The TODF peak shift, on the other hand, depends on the existence/non-existence of noise. The TODF peaks display *blue-shift* (no shift) in absence (presence) of noise [52] indicating enlargement of energy interval without noise as BE increases. The plot of peak value of TODF vs BE [Fig. 3d] shows persistent decrease with increase in BE under all conditions and complies with the previous findings.

## 4 Conclusion

Modulation of a few important NLO properties (EOE, TONOS and TODF) of doped QD by the variation of binding energy has been investigated. In this context the role of noise is thoroughly scrutinized. EOE and TONOS display steady fall as BE increases without noise and when MLN operates. However, in presence of ADN, above two NLO properties reveal persistent amplification with increase in BE. On the other hand, TODF decreases with increase in BE under all conditions. Thus, the rise/fall of NLO properties with BE depends on the existence/non-existence of noise, the noise mode and also on the particular NLO properties concerned. The pattern of peak-shift of the NLO properties with BE is conspicuously linked with the presence of noise although we do not find any remarkable role played by the noise mode. All the NLO properties invariably delineate blue-shift (no shift) with increase in BE when noise becomes absent (present). The observations bear importance in the study of NLO properties of LDSS when noise displays some crucial role.

## Declarations

### Author contribution statement

Manas Ghosh, Anuja Ghosh, Sk. Md. Arif: Conceived and designed the experiments; Performed the experiments; Analyzed and interpreted the data; Contributed reagents, materials, analysis tools or data; Wrote the paper.

### Funding statement

This research did not receive any specific grant from funding agencies in the public, commercial, or not-for-profit sectors.

### Competing interest statement

The authors declare no conflict of interest.

### Additional information

No additional information is available for this paper.
